# Evaluation of the effects of temperature and centrifugation time on elimination of uncured resin from 3D-printed dental aligners

**DOI:** 10.1038/s41598-024-66150-6

**Published:** 2024-07-02

**Authors:** Ji-Eun Kim, Utkarsh Mangal, Jae-Hun Yu, Gi-Tae Kim, Hoon Kim, Ji-Young Seo, Jung-Yul Cha, Kee-Joon Lee, Jae-Sung Kwon, Sung-Hwan Choi

**Affiliations:** 1 Department and Research Institute of Dental Biomaterials and Bioengineering, Yonsei University College of Dentistry, 50-1 Yonsei-ro, Seodaemun-gu, Seoul, Republic of Korea; 2 BK21 FOUR Project, Yonsei University College of Dentistry, 50-1 Yonsei-ro, Seodaemun-gu, Seoul, Republic of Korea; 3 Department of Orthodontics, Institute of Craniofacial Deformity, Yonsei University College of Dentistry, 50-1 Yonsei-ro, Seodaemun-gu, Seoul, Republic of Korea; 4 Research Institute of Agriculture and Life Sciences, College of Agriculture and Life Sciences, Seoul National University, Seoul, Republic of Korea

**Keywords:** Centrifugation, Uncured resin, Clear aligner, 3D printing, Cytocompatibility, Translucency, Orthodontics, Dentistry, Dental biomaterials

## Abstract

The study investigated the effects of temperature and centrifugation time on the efficacy of removing uncured resin from 3D-printed clear aligners. Using a photo-polymerizable polyurethane resin (Tera Harz TC-85, Graphy Inc., Seoul, Korea), aligners were printed and subjected to cleaning processes using isopropyl alcohol (IPA) or centrifugation (g-force 27.95*g*) at room temperature (RT, 23 °C) and high temperature (HT, 55 °C) for 2, 4, and 6 min. The control group received no treatment (NT). Cleaning efficiency was assessed through rheological analysis, weight measurement, transparency evaluation, SEM imaging, 3D geometry evaluation, stress relaxation, and cell viability tests. Results showed increased temperature and longer centrifugation times significantly reduced aligner viscosity, weight (*P* < 0.05), and transmittance. IPA-cleaned aligners exhibited significantly lower transparency and rougher surfaces in SEM images. All groups met ISO biocompatibility standards in cytotoxicity tests. The NT group had higher root mean square (RMS) values, indicating greater deviation from the original design. Stress relaxation tests revealed over 95% recovery in all groups after 60 min. The findings suggest that a 2-min HT centrifugation process effectively removes uncured resin without significantly impacting the aligners’ physical and optical properties, making it a clinically viable option.

## Introduction

The core principle of clear aligner therapy involves making slight adjustments to teeth positioning using a sequence of custom-made, transparent aligners^1^. The recent integration of clear aligner treatments into standard orthodontic practices is largely attributable to advancements in Computer-Aided Design/Computer-Aided Manufacturing (CAD/CAM) technology. The preferred method for creating these aligners has been to use thermoplastic materials, which are heated and then formed over three-dimensional (3D)-printed treatment stage models^2^. However, there have also been certain challenges noted, such as limited options for appliance customization, inconsistent quality among thermoplastic sheets, and less predictable treatment outcomes. Moreover, the single-use 3D-printed models that simulate the treatment stage for aligner production contribute to environmental concerns about adding to the generation of material waste^3,4^.

A recent advancement addressing the challenges outlined above involves directly 3D printing the aligners from CAD designs using clear resin materials^5^. This approach eliminates the need to produce an intermediate model, thus conserving time and labor, and it enables precise customization of the aligner dimensions to meet the specific anatomical needs of each patient. It also significantly diminishes the reliance on single-use polymeric treatment models^6^. The process follows the core principles of additive manufacturing for resin-based appliances and using vat-polymerization. The process is structured into three main phases: 3D scanning of the teeth, the additive printing process itself, and post-processing steps.

The post-processing stage, which occurs manually, entails the removal of any uncured monomer and ensures the aligner’s durability and safety through post-curing^7^. This stage is essential because the surfaces of appliances immediately after 3D printing often have excess uncured resin, which makes cleaning critical for biological safety. Most manufacturers recommend using an activated bath of organic solvents, such as ethanol or isopropyl alcohol (IPA), to clean the surfaces of 3D-printed appliances^8,9^. Although there are various opinions on the optimal IPA washing time, Jang et al.^7^ suggest that soaking in IPA for 1 min is clinically effective. However, the use of solvents that possess high volatility and flammability can lead to significant increases in airborne particles and total volatile organic compounds during the cleaning process^10^. Moreover, the chemical effects of these solvents on acrylic-based polymers can result in reduced translucency of clear resin material^11^. Organic solvents can also potentially exert unpredictable effects that alter the surface physical properties^9^. Therefore, with the sub-millimeter dimensional characteristic of the clear aligner, the cleaning method could potentially have a major influence on treatment outcomes. Hence, exploring non-chemical cleaning methods, such as centrifugation, for the removal of residual monomers has been proposed for 3D-printed clear aligner^12^.

The effectiveness of centrifugal resin removal can be influenced by factors such as the weight of the object, rotational speed, distance from the rotation axis to the object^13^, rotation time, viscosity^14^. Although previous studies have explored similar solvent-free centrifuge methods, the processes have been largely empirical, with minimal explanations of the key parameters^3,15^. Therefore, the objective of this study was to examine the effectiveness of an organic solvent-free centrifugal method for removing uncured resin from the surface of 3D-printed aligners. This investigation focused on the impacts of the temperature and the time of centrifugation on cleaning efficiency, and other parameters were kept constant. Herein, it was hypothesized that increases in the temperature and the duration of centrifugation influence the (i) weight, (ii) optical transparency, (iii) internal surface morphology, (iv) stress relaxation, and (v) cytotoxicity of the 3D-printed clear aligners.

## Methods

### Specimens preparation

**Figure 1:**
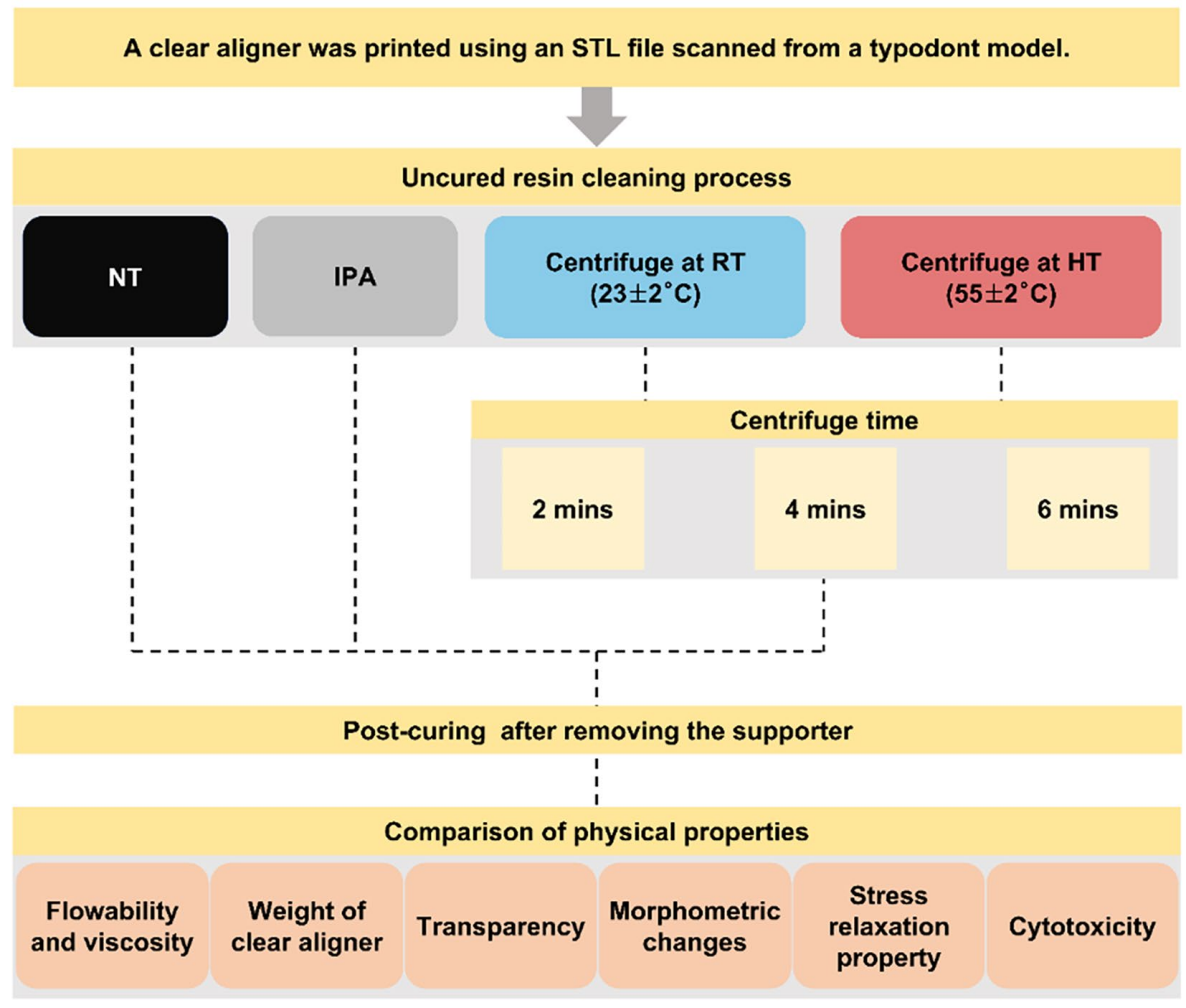
Flowchart depicting the study design and experiment workflow. NT; Not treated, IPA; Isopropyl alcohol, RT; Room temperature, HT; High temperature.

The study workflow is shown in Fig. 1. A typodont model representing maxillary dental arch (Dentiform; Nissan Dental Products, Kyoto, Japan) was used and scanned using an intraoral scanner (Medit i600, Medit Corp., Seoul, Korea) to create an Stereolithography (STL) file. Next, supports were added, and the aligners were designed with a predetermined thickness of 0.5 mm and an offset of 50 μm. The orientation of the models was set with the posterior section facing the platform at a 45° angle. They were then printed on the platform using an LCD 3D printer (UNIZ NBEE, UNIZ Technology LLC, USA) with a 49.8 µm XY resolution using a photo-polymerizable polyurethane resin (Tera Harz TC-85, Graphy Inc., Seoul, Korea).

**Figure 2:**
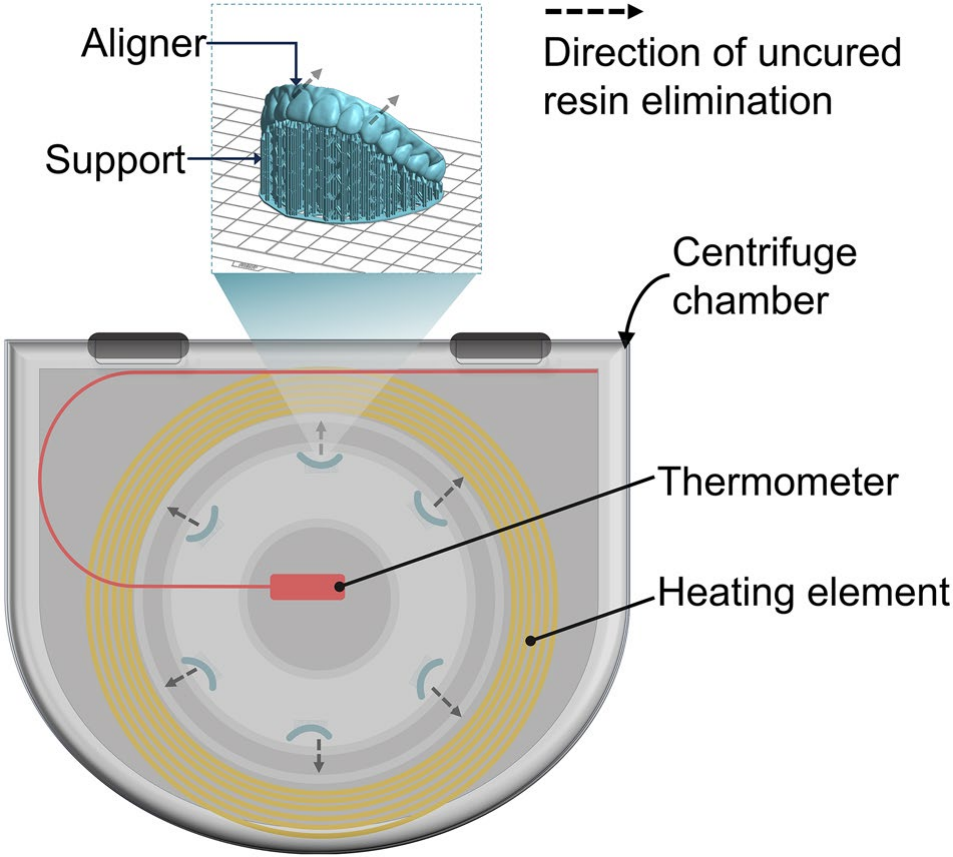
STL image of the printed clear aligner and schematic diagram of the centrifuge chamber with temperature control function.

After 3D printing, the aligners were separated from the build platform, and two different cleaning methods were used to remove uncured resin from the 3D-printed aligner: IPA and centrifuge cleaning. In the IPA group (negative control), the aligners were immersed in isopropyl alcohol (Sigma-Aldrich, 99.5%) and ultrasonic rinsed for 1 min using an ultrasonic cleaner (Sae Han Ultrasonic Co., Korea), and the aligners were placed on paper towels in the hood chamber to air dry for 5 min. The centrifuge group were treated using a centrifuge machine (MR-H100WM, Viska, Germany) with a heating element (YD07005-12002A, Wooheater Co., LTD, Korea) installed on the outside of the tub (Fig. 2). The temperature was controlled at 23 ± 2 °C (room temperature; RT) and 55 ± 2 °C (high temperature; HT). The HT setting used a temperature controller (OKE-6428HC, Sewon, Korea), and centrifugation (g-force 27.95*g*) was performed at 500 rotations per minute(rpm) for 2, 4, and 6 min at each temperature, respectively. The group that did not undergo any treatment is called NT (not treated). The supports were then removed from the aligners, and a 20-min curing process was performed under nitrogen using the Tera Harz Cure THC 2 UV curing system (Graphy, Seoul, Korea).

### Rheological analysis of 3D printing resin

Flowability and viscosity were measured to evaluate the flow properties of 3D printing resin at RT and HT (n = 5). For flowability, the resin solution was stored at RT and HT for 5 min each, after which 80 µL of the solution was dropped onto a glass slide and flowed tilted at 45° for 15 s to check the difference in flowability between the two temperatures. Viscosity tests were performed using an MCR 702e dynamic shear rheometer (Anton Paar, GmbH, Gratz, Austria) equipped with 25 mm parallel plates. The gap between rheometer plates was set to 1 mm. Resin viscosity was also measured at RT and HT and at shear rates ranging from 0.1 to 100 s^−1^.

### Weight of aligners after post-curing

After printing the clear aligner specimens, post-processing was performed for each group (n = 5) according to each condition. Next, the supports were carefully removed, and the clear aligners underwent post-curing. The weight of each manufactured model was then measured using a precision analytical balance (Metler Toledo ML104, Switzer). To minimize contamination during the support removal process and ensure consistent handling practices, fresh latex gloves were worn for each procedure.

### Cell viability

The cytotoxicity assessment was conducted while adhering to the protocols outlined in ISO 10993-5. The 3D-printed clear aligners of each group were cut into smaller specimens and sterilized using EO gas. The extracts of the specimens were immersed in RPMI 1640 culture medium (Welgene, Gyeongsangbuk-do, Korea) for 24 h at 37 °C, while maintaining a ratio of 0.2 g/mL (sample surface area/culture medium volume). The viability of L929 (ATCC, CCL-1, American type culture collection, San Diego, CA, USA) cultured with RPMI1640 cell culture medium as well as supplemented with 1% antibiotic–antimycotic (Gibco, Grand Island, NY, USA) and 10% fetal bovine serum (FBS; Gibco, Grand Island, NY, USA) was evaluated using the 3-(4,5-dimethylthiazol-2-yl)-2,5-diphenyltetrazolium bromide (MTT) assay. Cell viability was calculated as the percentage of optical density (OD) in comparison to the blank.

### Translucency measurement

The labial portion of the central incisor was positioned horizontally in front of the sample holder of a UV–visible spectrophotometer (Jasco UV–vis V-630, Tokyo, Japan) and aligned with the light source. Transmittance was measured in transmission mode for each sample. Transparency measurements for each sample (n = 3) were performed in the wavelength range from 350 to 750 nm. Each specimen was assessed with three consecutive measurements using the spectrophotometer, and the average value were calculated. The results were presented as area value (%) under the curve for each group computed using OriginPro software (OriginLab, OriginPro 8.5, USA) for transparency comparison. To visually compare the change in transparency the labial portion of the right central incisor from the clear aligner was isolated and photographed against two contrasting backgrounds: a neutral gray (RGB: 191, 191, 191) and a white background (RGB: 255, 255, 255).

### Surface topography analysis with scanning electron microscopy (SEM)

A representative specimen from each group was observed under a scanning electron microscope (JEOL JSM-IT-500HR, Tokyo, Japan). Before the SEM analysis, the specimen was platinum sputter coated for 90 s in a coating machine. The SEM micrographs were obtained at a magnification of 100× in vacuum, and an accelerating voltage of 15.0 kV.

### Morphometric analysis of aligner geometry

Plaster models were created by pouring gypsum (Mg Crystal Rock, Maruishi Gypsum Co. LTD., Osaka, Japan) into the support-attached printing models. Before the 3D superimposition, the cast models were scanned using a Model Scanner (Medit i600, Medit Corp., Seoul, Korea) to obtain 3D data for each experimental group (n = 3). The acquired scan data was saved in STL format, and a precision comparative assessment was conducted using a 3D morphometric program (Geomagic GmbH, Stuttgart, Germany) in the anterior region from canine to canine. Scanned data of the cast model (reference data) was overlaid with the typodont model data (standard data). After initial automatic alignment, the data sets were optimally aligned using Best Fit alignment, and the deviation for each specimen was calculated as the Root Mean Square (RMS) value. The RMS value was computed using the following formula:

1 $$RMS = \frac{{\sqrt {\mathop \sum \nolimits_{i = 1}^{n} \left( {X_{1,i} - X_{2,i} } \right)^{2} } }}{\surd n}$$

where $X_{1,i}$ is points measured from the Reference CAD data, $X_{2,i}$ is measured data from the specimen’s scan data, and $n$ is the total number of measurement points.

### Shape recovery from stress relaxation

For the stress relaxation test, specimens (n = 5) were printed with cylindrical shapes (diameter: 0.7 mm, height: 0.5 mm) added to both mesiobuccal cusp tips of the clear aligner model. The initial intermolar width (IM) was measured using a vernier caliper. Subsequently, the clear aligners from each group were immersed in boiling distilled water (DW) at 80 °C for 1 min. The specimens were bent in half at RT and placed in a mold (35 mm in width and 68 mm in length). The IM was immediately measured and recorded as 0 min, and the specimens were maintained in this position for 5 min. Afterward, the specimens were immersed in a water bath at 37 °C, and the stress relaxation phenomenon of the specimens was recorded at 10, 30, and 60 min.

2 $$Stress\;relaxation\;ratio = \frac{IM}{{initial\;IM}} \times 100\%$$

### Statistical analysis

Statistical analysis was performed using statistical software (IBM SPSS Statistics v27.0; IBM Corp. Armonk, NY). The non-parametric Kruskal–Wallis test was performed to compare differences in the weight, UV–vis transmittance, cytotoxicity and mean RMS values of the aligners, after which Mann–Whitney U tests were conducted for multiple comparisons. Results of stress relaxation test was performed using two-way ANOVA.

## Results

### Rheological analysis of 3D printing resin

**Figure 3:**
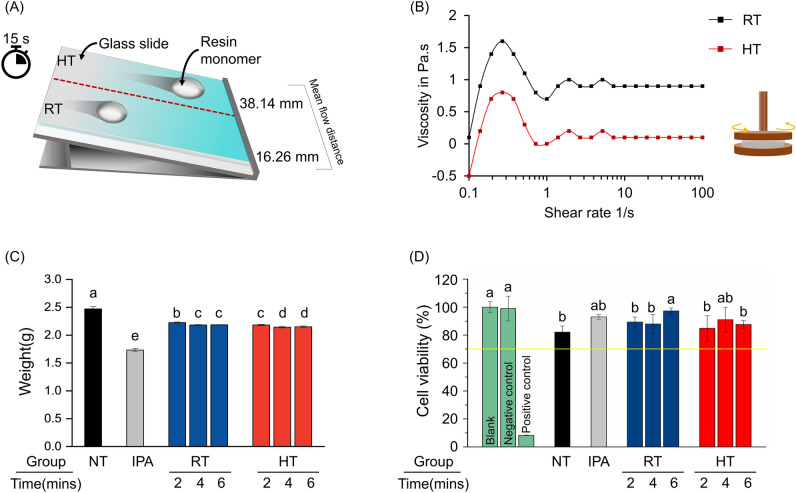
(**A**) Schematic diagram of the flowability experiment of solutions stored at each temperature. (**B**) Change in the viscosity as a function of shear force. (**C**) Average weight of 3D-printed aligner without supports according to the cleaning method used. (**D**) Cell viability of clear aligner model for each experimental group compared to the blank. The yellow line represents the minimum ISO standard (ISO 10993-5, 2009) criterion: a cell viability under 70% is cytotoxic. Different lowercase letters indicate significant differences in the average between groups (*P* < 0.05). NT, Not treated; IPA, Isopropyl alcohol; RT, Room temperature; HT, High temperature.

To measure flowability, the resin solution flowing down from the slide glass for 15 s was measured to be 16.26 ± 1.14 mm at RT and 38.14 ± 3.56 mm at HT, respectively (n = 5) (Fig. 3A). In the shear rate range from 0.1 to 100 s^−1^, the average value of RT was 0.95 Pa s and the average value of HT was 0.17 Pa s (Fig. 3B).

### Weight of aligners after post-curing

The average weight of the post-cured aligner samples, expressed as mean ± SD, for the groups were as follows: NT (2.47 ± 0.04 g), IPA (1.73 ± 0.03 g), RT-2 (2.23 ± 0.01 g), RT-4 (2.18 ± 0.07 g), RT-6 (2.19 ± 0.05 g), HT-2 (2.18 ± 0.01 g), HT-4 (2.14 ± 0.02 g), and HT-6 (2.15 ± 0.02 g). The average weight was significantly higher in the NT group and significantly lower in the IPA group compared to the other groups examined (*P* < 0.05). There was a significant difference in weight depending on temperature and time, but there was no significant difference between RT-4, 6 min and HT-2 min, and between HT-4 and 6 min (Fig. 3C).

### Cell viability

Figure 3D presents the cell viability data for various tested groups. The cell viability values are as follows: the positive control at 8.35 ± 0.20%, the negative control at 98.99 ± 8.78%, the NT group at 82.10 ± 4.46%, and the IPA group at 92.98 ± 1.64%. For the RT-2, RT-4, and RT-6 groups, the values are 89.31 ± 3.61%, 87.90 ± 6.80%, and 97.26 ± 2.06%, respectively. Similarly, the HT-2, HT-4, and HT-6 groups have cell viability values of 84.88 ± 9.04%, 91.02 ± 8.90%, and 87.61 ± 2.77%, respectively. Notably, the RT-6 and HT-4 groups exhibited significantly higher cell viability compared to the other experimental groups (P < 0.05).

### Translucency measurement

**Figure 4:**
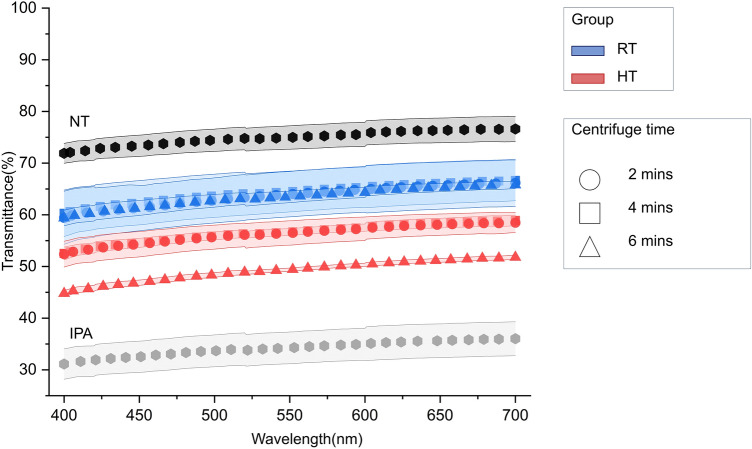
Transmittance curves of all groups of clear aligners with SD. NT, Not treated, IPA, Isopropyl alcohol; RT, Room temperature; HT, High temperature.

The calculated areas under the transmittance curves for each cleaning method presented transmittance (%) in descending order: NT (74.64%), RT-2 (63.84%), RT-4 (63.19%), RT-6 (63.00%), HT-2 (56.12%), HT-4 (55.87%), HT-6 (48.73%), and IPA (33.99%), as shown in Fig. 4. RT exhibited higher transmittance compared to HT, with RT-2, RT-4, and RT-6 showing consistently higher values than their HT counterparts. Transparency tended to decrease with increasing centrifugation time within both the RT and HT groups; however, only HT-6 demonstrated a significant (*P* < 0.05) reduction in transmittance.

**Figure 5:**
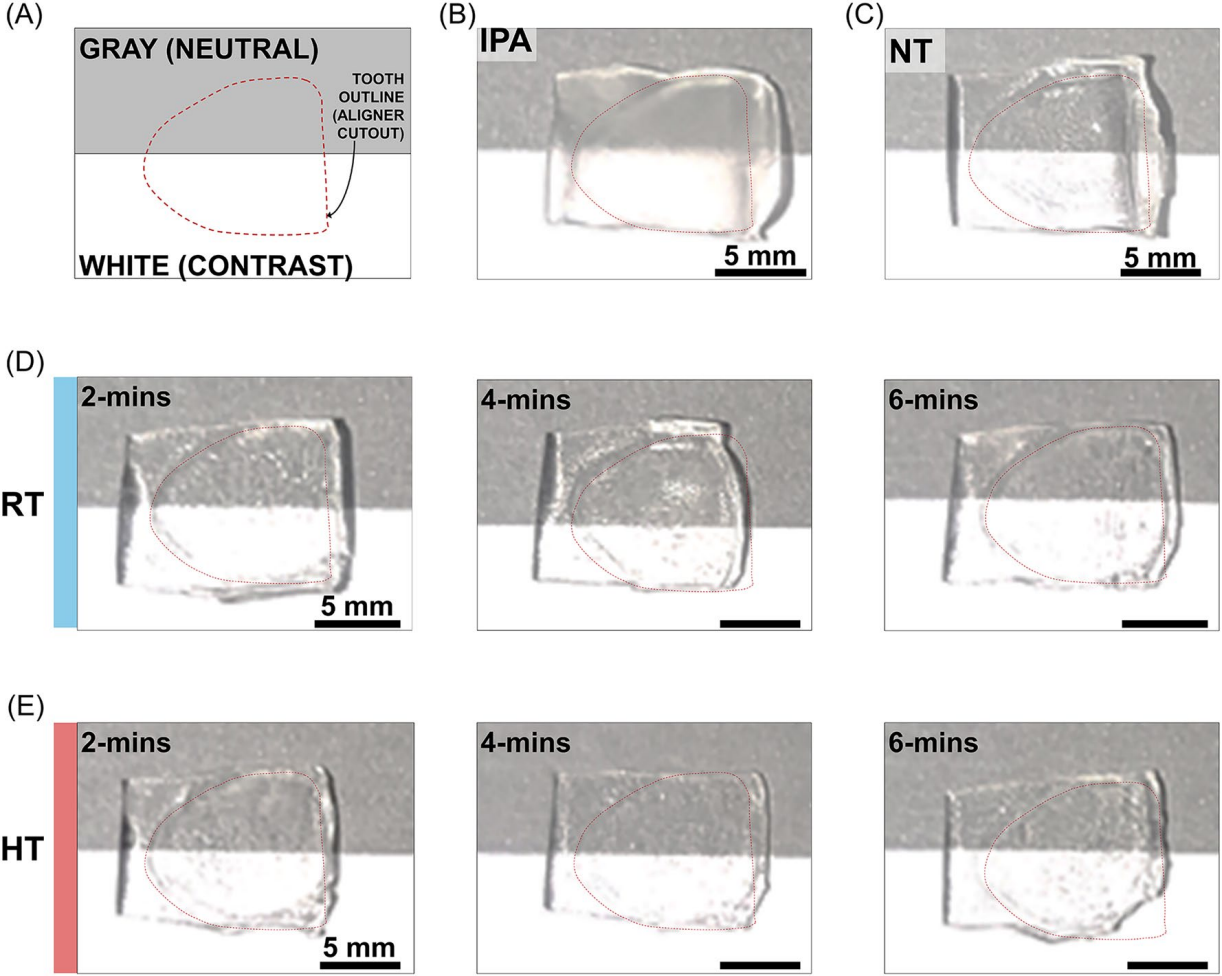
Comparison of translucency on gray and white backgrounds by cropping the right maxillary central incisor region from each group’s specimens. (**A**) schematic diagram showing sample alignment for imaging, (**B**) IPA, (**C**) NT, (**D**) RT-2, 4, 6 min, and (**E**) HT-2, 4, 6 min. NT, Not treated; IPA, Isopropyl alcohol; RT, Room temperature; HT, High temperature.

In visual observation, comparing the transparency in the two contrasting backgrounds (Fig. 5A), IPA showed hazy dull appearance compared to the other groups (Fig. 5B). There were no differences found between the NT (Fig. 5C), RT (Fig. 5D) and HT (Fig. 5E) groups.

### Surface topography analysis with SEM

**Figure 6:**
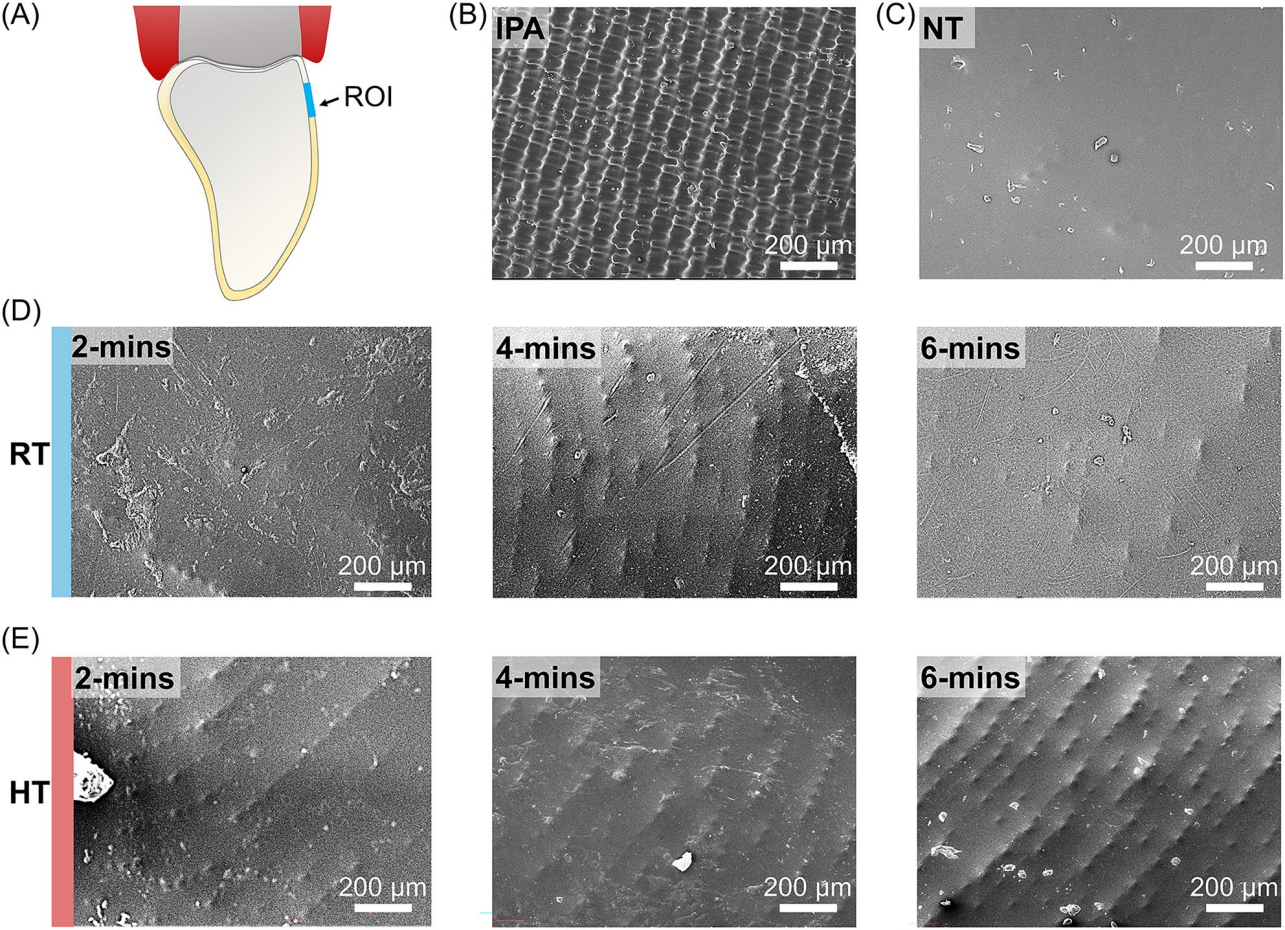
SEM images (×100 magnification) of clear aligners. (**A**) A schematic diagram of the SEM image capture locations, (**B**) IPA, (**C**) NT, (**D**) RT-2, 4, 6 min, and (**E**) HT-2, 4, 6 min. ROI, region of interest; NT, Not treated; RT, Room temperature; HT, High temperature; IPA, Isopropyl alcohol.

Figure 6A shows the SEM images of different post-processed specifications at 100 × magnifications with region of interest in labial gingival third. The IPA group showed the most uneven surface, but it appeared to have a consistent pattern (Fig. 6B). No layer was observed in the NT (Fig. 6C) and RT-2 min group, and the NT group showed the smoothest surface. SEM images of RT-4, 6 min (Fig. 6D) and HT-2, 4, 6 min (Fig. 6E) groups showed similar surface morphology, and the printed layer could clearly be observed. Among them, the printed layer of the specimen of the HT-6 min group was most clearly observed.

### Morphometric analysis of aligner geometry

**Figure 7:**
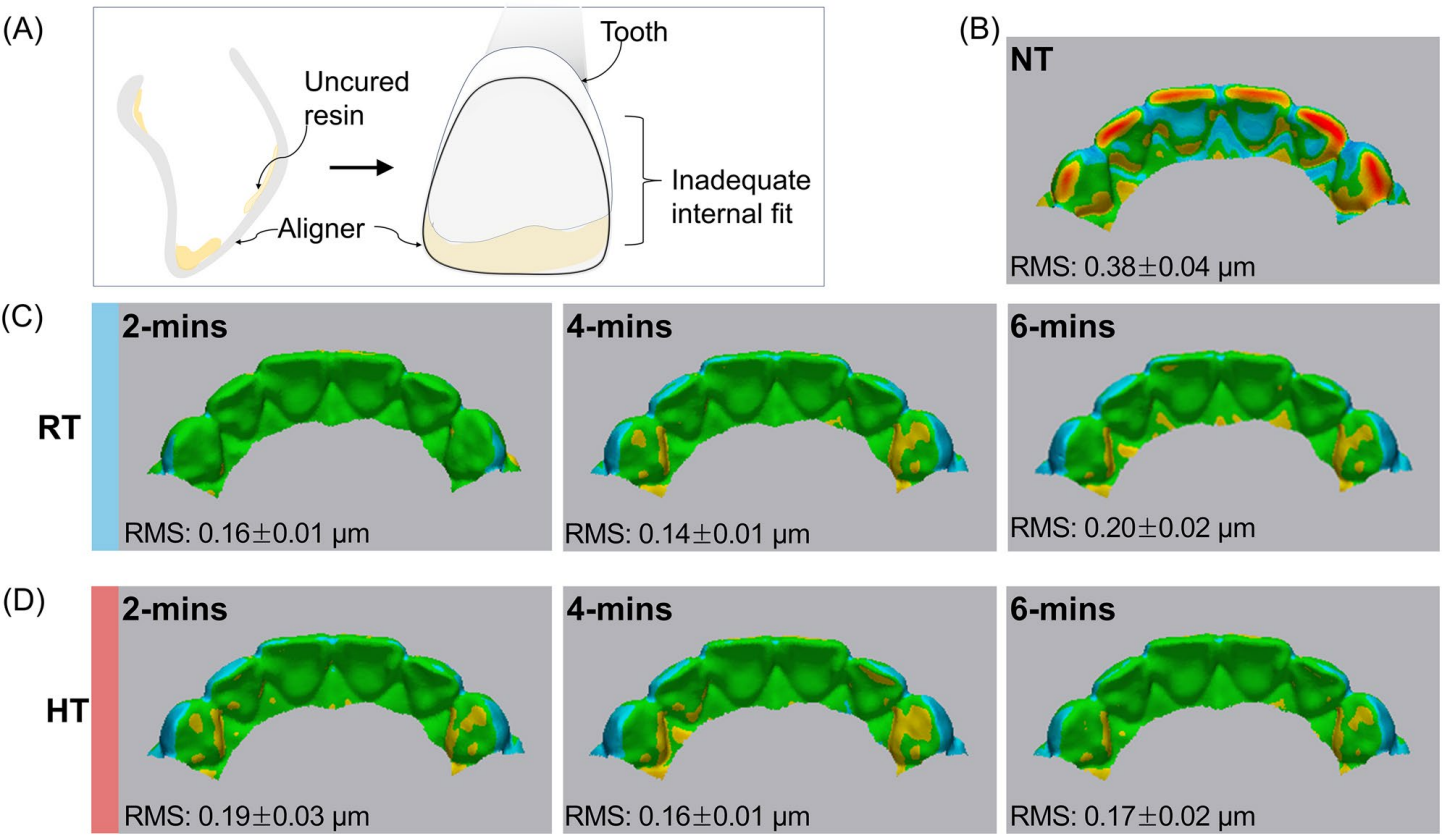
Comparison of color difference maps of anterior region evaluated using Geomagic software program. (**A**) a schematic diagram illustrating the residual monomer pattern on the printed clear aligner before the cleaning process; (**B**) NT; (**C**) RT-2, 4, 6 min; (**D**) HT-2, 4, 6 min. NT, Not treated; RT, Room temperature; HT, High temperature; RMS, root-mean square value.

This study used a color-difference map to qualitatively compare the effect of inadequate uncured resin removal on fit of clear aligners (Fig. 7A). The index of the color-difference map was set to ± 100 μm, and the differences between the two groups were expressed as color differences. Positive (+) deviations are shown in red, the closer the deviation is to 0, the more the color is green, and negative (−) deviations are shown in blue. As a result, the distribution of color-difference maps was similar aside from the NT group. In the NT group, positive deviations were prominent with larger red areas appearing in the anterior incisal region (Fig. 7B), while no red areas were observed at all in the other groups. RT-2 min were within the tolerance range (green color). Yellow color appeared on the lingual part of the canine tooth in the RT and HT groups, aside from the RT-2 min (Fig. 7C, D). In terms of RMS value, the NT group had a significantly larger RMS value (*P* < 0.05) compared to the other groups, while there was no significant difference in the RMS values among the other groups.

### Shape recovery from stress relaxation

All groups showed an increasing trend in the shape recovery ratio over time (Table 1). Upon 60 min of relaxation all groups exhibited a shape recovery rate of over 95%. There were no statistically significant differences between groups, however after 30-min the HT groups showed a marginally higher percentage shape recovery compared to the RT groups.

**Table 1:** Shape recovery ratio of clear aligners specimens (%).

Duration (mins)	NT	RT-2	RT-4	RT-6	HT-2	HT-4	HT-6
0	48.29 ± 2.98^Aa^	45.86 ± 1.32^Aa^	44.28 ± 2.69^Aa^	44.00 ± 2.72^Aa^	46.44 ± 2.90^Aa^	48.26 ± 2.11^Aa^	46.81 ± 1.87^Aa^
10	79.74 ± 5.66^Ab^	81.6 ± 9.33^Ab^	75.64 ± 9.91^Ab^	83.44 ± 5.41^Ab^	79.10 ± 9.73^Ab^	80.32 ± 8.91^Ab^	74.88 ± 3.61^Ab^
30	92.18 ± 2.49^Ac^	90.82 ± 4.26^Ac^	91.74 ± 3.73^Ac^	93.35 ± 3.73^Ac^	95.32 ± 1.87^Ac^	94.91 ± 5.11^Ac^	92.91 ± 3.09^Ac^
60	96.80 ± 1.01^Ad^	96.93 ± 2.94^Ad^	95.91 ± 1.21^Ad^	98.79 ± 1.77^Ad^	99.23 ± 1.16^Ad^	99.37 ± 3.82^Ad^	98.70 ± 4.17^Ad^

Same capital letters indicate no significant differences in the average between groups at the same minutes. Different lowercase letters indicate significant difference between relaxation time within the same group(*P* < 0.05).

## Discussion

The present study investigated the temperature and time of a centrifugation cleaning method for the removal of uncured monomer fraction from 3D-printed clear aligners, while focusing on their surface characteristics and optical features. The results demonstrated that, while increases in temperature and time did not alter the surface properties, it affected translucency and the internal surface morphology of the aligners.

Recent advancements in clear aligner production have leveraged additive manufacturing due to its high precision and ease of production. Given the importance of aligners’ physical and optical qualities, an organic-solvent-free centrifugation method has been suggested as a method that could be used for the effective removal of any uncured resin. Although a basic centrifugation protocol is established, knowledge of the most effective settings primarily relies on empirical evidence. To explore the optimal physical conditions for effective centrifugation, we modified centrifugal equipment to operate at a standard RPM and added a heating element around the chamber’s edge. We carefully ensured that the heating element avoided contact with the rotating chamber to preserve rotation efficiency (Fig. 2). We achieved temperature control using a digital thermostat, and cycle durations were adjusted manually. Moreover, for efficient centrifugation, aligners were loaded in a balanced manner in each cycle.

To characterize the basic reaction of the supplied resin to heat, we conducted experiments to observe its viscosity and flow. The resin exhibited a predictable increase in flow when heated, which was consistent with viscosity profiles that are typical of most liquid-type resins at elevated temperatures^16^. There is also a direct correlation between the viscosity of a liquid and its flow rate. In a solvent-free centrifuge setup, this relationship could play a crucial role in effectively removing the uncured resin fraction^14^. In this study, the 3D printing resin demonstrated an approximately 2.34-fold increase in flow upon heating to 55 °C, which ultimately resulted in increased flowability and decreased viscosity. When evaluating the viscosity change under both temperature and physical shear force, the clear aligner resin exhibited a significant reduction in viscosity, approaching zero Pascal-seconds. This finding further supports the idea that centrifugal cleaning effectiveness can be enhanced by increasing the chamber temperature.

Transparency in clear aligner is the key to the aligner’s clinical compliance and treatment success^17^. In this study, the IPA group, which had a significantly lower weight, also exhibited significantly lower transparency. This finding aligns with another study that demonstrated significantly lower transparency in the IPA group compared to the centrifuge cleaning method. Additionally, the IPA cleaning group had thinner specimens compared to the centrifuge cleaning method group^11^. Combining these findings, there appears be a correlation between the weight, thickness, and transmittance of 3D-printed clear aligners; lighter aligners tend to be thinner, and thinner aligners may exhibit lower transmittance. In this study, the weight of the aligners decreased in the following order: NT > RT-2 > RT-6, RT-4, HT-2 > HT-6, HT-4 > IPA. Interestingly, the UV–vis transmittance followed a similar trend, with the groups ordered as NT, RT-2, RT-4, RT-6, HT-2, HT-4, HT-6 > IPA. However, the relationship between weight and transparency was not perfectly proportional. This could be attributed to the limited number of specimens tested or the possibility that the differences in thickness were not substantial enough to significantly impact the transmittance.

The optical properties of materials are determined by how they respond to light in terms of absorbance and transmittance. The higher the transmittance of material, the more transparent it is; conversely, higher absorbance indicates lower transparency^18^. Contrary to our findings, which suggest a proportional relationship between thickness and transmittance, the Beer-Lambert law (T = exp(− μ_a_*d*), where T is the transmittance, *d* is the sample thickness, and μ_a_ is the absorption coefficient) states that for transparent, non-scattering medium, transmittance is inversely proportional to sample thickness^19^. These results may be related to the surface morphology of the material. It has been demonstrated that, beyond the rinsing solution, the time and method also significantly affect the surface morphology and roughness of materials^3,9^. The findings of the present study also add to this evidence corroborating that chemical and non-chemical cleaning techniques differentially affect the surfaces of 3D-printed aligners. Analyzing the SEM images alongside transmittance data, it could be observed that the IPA group, which displayed the lowest levels of light transmittance, had the most irregular surface texture. Conversely, the NT group surface was considerably smoother compared to those of the other groups.

The findings of our study are consistent with these observations. Moreover, SEM revealed noticeable differences in the inner surface of the aligners with increasing temperature. These results may be related to the measurements of the aligner weight, suggesting that the more clearly the layer is visible on the transparent aligner surface, the less monomer remains. This implies that the removal of uncured resin reveals the micro-surface fingerprints characteristic of 3D printing^20^. Such exposure could indirectly affect light transmission and, in turn, influence the translucency of the aligners, regardless of their thickness. Furthermore, while no significant changes were observed in the net weight of the aligners, variations in translucency were noted with changes in temperature.

In the group where layering was evident in the SEM images, the inter-layer spacing appeared to exceed 50 µm, thus diverging from the predefined layer thickness settings during the printing process. This discrepancy arises because the specimens in this study were not produced as flat shapes but rather as three-dimensional aligners. Further, clear aligners printed at 45 degrees have intermediate options between horizontal and vertical orientations in terms of print layers, print time, and space taken up on the build platform, so these parts are printed at an oblique angle^21^. The choice to photograph the cervical third of the aligner was deliberate, as this area is most distinct and offers a high contrast between the gum tissue and tooth; this is particularly noticeable when people smile or talk^22,23^. Enhancing the printing resolution, specifically the layer proximity, might mitigate this effect, which is a relationship that might require further research.

3D-printing resins are highly cytotoxic prior to the 3D-printing process, and cytotoxic levels are significantly reduced after post-polymerization procedures to remove uncured resin^24^. In this study, there was a statistically significant difference in the cell viability values between experimental groups; however, there were no trends with increasing time or temperature. The NT group showed the lowest cell survival rate among the experimental groups. All groups were found to comply with ISO 10993-5 standards. This suggests that it is biocompatible for oral use regardless of the cleaning method.

The arch-width at the intermolar region is a dynamic value that changes significantly with an individual’s development. In orthodontic treatment, avoiding any unwarranted reduction in intermolar width (IM) is crucial for outcome stability. By analyzing the stress relaxation-driven shape recovery, we confirmed that there was no unwarranted reduction in width, with over 95% recovery observed after 60 min. This finding is consistent with previous studies that demonstrated an increase in recovery rate over time during bending tests. This suggests that the centrifugation cleaning temperature and time do not significantly compromise the shape memory properties of clear aligners, which are crucial for their clinical performance and treatment outcomes^25^.

Lastly, we assessed the aligner adaptation accuracy by comparing the internal surface morphology to the intended design. The quality of the aligner fit to the tooth surface significantly impacts the delivery of force necessary for effective orthodontic tooth movement^21,26^. Given that aligners cover all exposed anatomical surfaces of a tooth, any misalignment can substantially disrupt the intended tooth movement. Misalignments could also result in premature occlusal interference, ultimately leading to discomfort and potential adverse effects^27^.

Considering the poor translucency, the IPA group was excluded from the internal fit assessment. The differences among the centrifugation groups were qualitatively evaluated using color map analysis. In this analysis, a yellow–red gradient indicates an area where the aligner thickness has increased non-uniformly internally, due to aggregation of uncured resin post-centrifugation. Meanwhile, a cyan to blue gradient an area where the surface has become thinner. To provide a baseline for comparison, a morphometric analysis of the NT group was performed. As seen in the 3D color map difference analysis in Fig. 7A inefficient resin removal adversely affects the incisor edge which is a geometrically tapered region. An aligner experiencing this issue is likely to fails in properly engaging the tooth surface, which can lead to an unintentional increase in the incisal dimension or complete loss of fit. This, in turn, may cause severe discomfort.

The centrifugation groups did not exhibit statistically significant differences in RMS values with variations in temperature and time. However, qualitative assessments revealed well-aligned incisal edges for both HT and RT conditions at 2 min. Further, the adaptation at the cingulum surface on the palatal side toward the mesial line angle of the canine showed a sporadic yellow gradient. This indicates that the arch’s curvature might affect the efficiency of removing uncured monomer following 3D printing. A previous study that compared internal fit also identified a potential gap near the cingulum surface in the gingival third of the anterior region^11^. Although they used micro-computed tomography for their assessment, their findings are consistent with those of the current study. Moreover, the RMS value was derived by creating a 3D model from the internal surface of the aligner, essentially using it as a mold. Given the indirect nature of this assessment method, objectively generalizing the RMS results presents a challenge. In addition, the absence of a definitive, practical guideline for evaluating clear aligner fit complicates the achievement of a high-precision methodology. Despite these challenges, the methodology employed is detailed in such a manner that supports reproducibility in future research.

Despite the comprehensive findings, this study has several limitations. Firstly, there are currently no standardized indicators or benchmarks to define the transparency of clear aligners. As a result, this study could only conduct a relative evaluation of transparency rather than an absolute one. Additionally, a clear aligner produced by scanning a standardized typodont model was used in this study. In clinical practice, clear aligners are prescribed for patients with varying magnitude of malocclusion. Therefore, further research is needed to determine generalization of the proposed cleaning protocol when applied to clear aligners for patients with severe crowding.

Moreover, this study has broader implications beyond orthodontic clear aligners. Recently, the centrifugal cleaning method has been employed in dentistry to produce temporary 3D-printed fixed dental prostheses. Research on centrifugal cleaning using various 3D printing materials has shown that, unlike mechanical cleaning, chemical cleaning impairs flexural strength, and the cleaning strategy recommended by individual manufacturers does not always result in the highest mechanical properties for each material^3^. Although our study primarily focused on clear aligners, the results may have a wide-ranging impact across dental restorative treatments. We hope that the findings of this study will contribute to the advancement of dental 3D printing technology and the expansion of its clinical applications.

## Conclusion

The results of the present study demonstrated that the centrifugation cleaning method is effective for removing uncured resin from clear aligners immediately after 3D printing. Increasing the temperature to 55 °C during centrifugation enhanced resin removal by reducing its viscosity and improving flow. However, centrifugation at this temperature for more than 2 min negatively impacted the translucency of the aligners. Neither the temperature nor the duration of centrifugation cleaning significantly affected the optical properties, cell viability, or stress relaxation properties of the aligners. Based on these findings, it is recommended that centrifugal cleaning at 55 °C for 2 min with a force of 27.95 g effectively removes uncured resin from aligners while maintaining clinically desirable esthetics.

## Data Availability

Data will be made available from the corresponding author on reasonable request.
